# Awareness of Alcohol and Cancer Risk and the California Proposition 65 Warning Sign Updates: A Natural Experiment

**DOI:** 10.3390/ijerph191911862

**Published:** 2022-09-20

**Authors:** Alexandra Budenz, Richard P. Moser, Raimee Eck, Tanya Agurs-Collins, Timothy S. McNeel, William M. P. Klein, David Berrigan

**Affiliations:** 1 Tobacco Control Research Branch, Behavioral Research Program, Division of Cancer Control and Population Sciences, National Cancer Institute, 9609 Medical Center Drive, Rockville, MD 20850, USA; 2 Office of the Associate Director, Behavioral Research Program, Division of Cancer Control and Population Sciences, National Cancer Institute, 9609 Medical Center Drive, Rockville, MD 20850, USA; 3 Health Behaviors Research Branch, Behavioral Research Program, Division of Cancer Control and Population Sciences, National Cancer Institute, 9609 Medical Center Drive, Rockville, MD 20850, USA; 4 Information Management Services, Inc., 3901 Calverton Blvd #200, Calverton, MD 20705, USA

**Keywords:** alcohol, cancer prevention, policy, point of sale warnings, awareness

## Abstract

In 1986, California enacted Proposition 65 (P65), requiring businesses to display warning signs informing consumers that specific chemicals and alcohol exposure increase the risk of cancer and reproductive harm. In 2018, the P65 alcohol warning signs were updated to include an informational P65 website link, and the update was associated with media coverage and increased enforcement of warning requirements. This study examines knowledge of the association between alcohol use and cancer risk in California compared to the rest of the US before and after the 2018 P65 update. We analyzed state-level data on alcohol and cancer knowledge from the Health Information National Trends Survey from 2017 (n = 3285), 2019 (n = 5438), and 2020 (n = 3865). We performed multinomial logistic regressions to examine knowledge levels by survey year and location (California vs. all other states) and reported the predicted marginals of knowledge by survey year and location. The adjusted prevalence of respondents who reported an association between alcohol and cancer risk was higher in California (41.6%) than the remaining states (34.1%) (*p* = 0.04). However, knowledge levels decreased significantly over survey years, and there was no evidence for an effect of the P65 update on knowledge in California compared to other states based on the testing of an interaction between state and year (*p* = 0.32). The 1986 warning signs may have had an enduring effect on awareness, though the update, so far, has not. Further efforts are needed to determine how to increase alcohol and cancer knowledge to address the burden of alcohol-attributable cancers.

## 1. Introduction

In 2011–2015 the number of annual deaths from excessive alcohol consumption in the US was estimated to average 93,296 [1]. Alcohol is a risk factor for multiple cancer types, including esophageal, colon, and breast cancers, and recent studies estimate that 3–4% of all cancer deaths are attributable to alcohol [2,3,4]. Research has demonstrated that there is no safe level of alcohol consumption as it pertains to cancer risk, and there is a dose-response relationship between consumption and risk of some cancers including breast [5].

Despite extensive research establishing alcohol as a cancer risk factor, knowledge about alcohol use and cancer risk in the US is relatively low. One study using the 2017 Health Information National Trends Survey (HINTS) found that only 38% of US adults (18 years or older) surveyed knew of the association between alcohol and cancer [6]. The 2019 American Institute for Cancer Research Cancer Risk Awareness Survey found that only 45% of US adults (18 years or older) were aware that alcohol is a cancer risk factor [7]. In addition to increasing the probability of healthy behavior related to alcohol, awareness of the association between alcohol and cancer is associated with support for alcohol control policies [8,9].

One approach to increasing alcohol and cancer knowledge is the use of alcohol warnings displayed at the point of sale. Point-of-sale warnings are typically required for both on-premises (e.g., bars) and off-premises (e.g., liquor store) alcohol retailers and are often posted by the front door of an establishment, near the register, near shelves on which alcohol is displayed, or behind a bar [10]. The goal of these warnings is to promote informed decisions about alcohol consumption by exposing potential consumers to risk information prior to purchasing an alcohol product [10,11,12], and therefore reduce alcohol consumption and alcohol-attributable health conditions [13]. Twenty-three US states and DC have laws requiring point-of-sale alcohol warnings about the risks of alcohol consumption during pregnancy; placement of the warnings and retailers required to display warnings varies by state [10,14]. Note that these warnings do not mention cancer risk. Point-of-sale warnings in certain states complement the existing 1989 federal law requiring alcohol warning labels with information about alcohol use during pregnancy and while operating heavy machinery or vehicles be affixed to all alcohol containers [11].

Few studies evaluating point-of-sale alcohol warnings have been conducted in the US; those that have been conducted focused on alcohol consumption during pregnancy [10,14,15]. These studies found associations between point-of-sale alcohol warning sign laws and decreased odds of any alcohol use [10], binge drinking [14] among pregnant women, and increased knowledge about the risks of drinking during pregnancy [15], compared to states without such laws. Evidence is mixed for the effectiveness of warning labels on alcohol packaging itself.

Studies have also examined the effects of point-of-sale health warnings for sugary drinks and tobacco products. One study of point-of-sale sugary drink warnings found that participants in a simulated shopping task study exposed to pictorial warnings displayed alongside a display of drinks were less likely to select sugary drinks than those who were unexposed [16]. Studies of point-of-sale tobacco warnings (displayed at cash register, entrance of establishment, or shelves on which tobacco products were displayed) have also shown that graphic warnings may increase awareness about smoking risks [17], negative affective responses to tobacco products [18], and interest in quitting smoking [19]. However, we are unaware of population-level point-of-sale alcohol warning studies in the US that assess knowledge about alcohol and cancer risk.

In 1986, California enacted Proposition 65 (P65), a state-level policy requiring businesses to display warning signs informing consumers that exposure to certain chemicals, including alcohol, increases the risk of cancer and reproductive harm [20]. This proposition is unique to California; no other US states mandate such warnings. The alcohol-specific P65 regulations were updated to require the addition of the phrase “For more information go to www.p65warnings.ca.gov/products/alcoholic-beverages” (accessed on 13 September 2022) to the pre-existing required text, in addition to expanding this warning requirement to include online vendors. This update was adopted in 2016 but was not made operative until August 2018 (https://www.p65warnings.ca.gov/businesses/new-proposition-65-warnings, accessed on 13 September 2022). At the website, consumers can find in-depth information about the alcohol-cancer link. The changes were associated with media coverage (including attention in special interest publications and some in general news outlets but no formal media campaign) and an increase in the enforcement of warning requirements, thereby potentially increasing awareness of the alcohol-cancer link.

This study examined knowledge of the association between alcohol use and cancer risk in California versus the rest of the US. First, we assessed the awareness of alcohol as a cancer risk factor over a three-year period (2017–2020), hypothesizing that California would exhibit higher awareness than the rest of the US. Second, because data were collected before and after the 2018 P65 update, we also assessed whether differences in awareness between California and the US changed between 2017 and 2020.

## 2. Materials and Methods

We analyzed data from three HINTS iterations: 2017 (HINTS 5 cycle 1; n = 3285; response rate = 32.4%), 2019 (HINTS 5 cycle 3; n = 5438; response rate = 30.3%), and 2020 (HINTS 5 cycle 4; n = 3865; response rate = 36.7%). After excluding respondents who had missing data for any of the variables used in the analysis (16.1%), the final study sample was n = 10,562. HINTS is a probability-based, nationally representative survey administered by the National Cancer Institute. The population for HINTS includes US civilian, noninstitutionalized adults (≥18 years-old). HINTS 5 cycles 1 and 4 were sent by mail and self-administered, and HINTS 5 cycle 3 used a mixed-mode option of either self-administered or online administration. Further details on the sampling and weighting procedures are publicly available (http://hints.cancer.gov (accessed on 13 September 2022)). HINTS survey items are selected from trusted, existing surveys and/or developed and modified to address specific questions related to cancer-related information or knowledge. All new HINTS survey items are subject to rigorous cognitive interviewing and pilot testing [21,22]. Each HINTS dataset contains a final sample weight to obtain population-level point estimates and jackknife replicate weights to obtain accurate variance estimates. The secondary analysis of HINTS without personal identifiers data is considered “not human subjects research” by the National Institutes of Health and did not require IRB review.

### 2.1. Survey Items

#### 2.1.1. Outcomes: Knowledge of Alcohol and Cancer Association

In HINTS 5 cycles 1 and 3, participants were asked, “Which of the following health conditions do you think can result from drinking too much alcohol?” and provided a list of health conditions, including cancer. The response options for each condition were, ‘Yes’, ‘No’, or ‘Don’t know.’ In HINTS 5 cycle 4, there was a change to the wording of the alcohol and cancer knowledge item; participants were asked, “In your opinion, how much does drinking the following types of alcohol affect the risk of getting cancer?” and listed beer, wine, and liquor as discrete items. This change was made to explore knowledge and attitudes concerning the three different types of alcohol as beverage type is often viewed as a potential influence on the health effects of alcohol [23]. For each type, participants could respond, ‘Decreases risk a lot’, ‘Decreases risk a little’, ‘No effect’, ‘Increases risk a little’, ‘Increases risk a lot’, and ‘Don’t know.’

To harmonize data across cycles, we recoded the cycle 4 responses to produce results that conform to the item in cycles 1 and 3 (i.e., Yes, No, and Don’t Know). For participants who responded that they believed that any of the three types of alcohol ‘Increase risk’, the response was recoded to ‘Yes.’ If a participant responded with any combination of ‘No effect’ or ‘Decreases risk’ to at least one alcohol type and responded, ‘No effect’, ‘Decreases risk’, or ‘Don’t know’ to the others, the response was recoded to ‘No’. If a participant responded ‘Don’t know’ across all alcohol types, the response was recoded as ‘Don’t know’. For any combination of ‘No effect’, ‘Decreases risk’, and additional missing responses or missing responses for all alcohol types, the variable was coded as missing.

Our recoding decisions reflect the results of a preliminary analysis of the consistency in responses across alcohol types. Across the knowledge response options we found that most participants answered consistently to at least two of the three alcohol types (e.g., over 90% of those who responded ‘Yes’ responded consistently across at least two alcohol types). These results suggest that our recoding appropriately reflects knowledge and beliefs about alcohol and cancer.

#### 2.1.2. Locations for Comparison

We merged restricted HINTS data that contained the state of residence of each respondent to compare alcohol and cancer knowledge in California versus the rest of the US (aggregate of the remaining states, excluding California). State of residence is not typically included in public HINTS datasets but can be requested through a special data use agreement. Sample sizes per cycle ranged from 348–612 in California and 2394–3867 in the remaining states.

#### 2.1.3. Alcohol Consumption

Alcohol consumption data were available only in HINTS 5 cycle 4 and were only used for descriptive analyses. An alcohol use variable with the categories ‘non-drinker’ (no drinking in the past 30 days), ‘some drinking’, and ‘risky drinking’ was created. Participants who reported binge and/or heavy drinking in the past 30 days (i.e., females who reported consuming more than seven alcoholic beverages/week and males who reported consuming more than 14 alcoholic beverages/week) and/or past-30 day binge drinking (females who reported consuming four or more alcoholic beverages on one occasion and males who reported consuming five or more alcoholic beverages on one occasion) were categorized as ‘risky drinkers’ [24]. Participants who reported drinking alcohol at least one day per week but did not meet the criteria for ‘risky drinking’ were categorized as ‘some drinking’.

#### 2.1.4. Covariates

Other variables included as covariates in the analysis were: ‘gender identity’ (female, male), ‘age’ (18–39 years, 40–59 years, 60+ years), ‘education’ (high school degree or less, post high school training or some college, college graduate or more), ‘race’ (Black, White, other/multiple (included American Indian/Alaska Native, Asian, Native Hawaiian/other Pacific Islander, and multiple races- combined due to small sample sizes)), ethnicity (Hispanic, not Hispanic), ‘sexual orientation’ (heterosexual, homosexual/gay/lesbian, bisexual), ‘personal cancer history’ (Yes, No), ‘family cancer history’ (Yes, No), and ‘cigarette/e-cigarette use’ (Current user/Non-current user), which was included because tobacco and alcohol use often co-occur. Personal and family cancer histories were included because cancer knowledge in people who have experiences with cancer may differ from knowledge in the general population. To assess cigarette or e-cigarette use, participants were asked, “How often do you now smoke cigarettes?” (Every day, Some days, Not at all) and “Do you now use an e-cigarette every day, some days, or not at all?”. Participants who reported using cigarettes or e-cigarettes every day or some days were categorized as current smokers or e-cigarette users. All others were considered non-current users. Other tobacco product use (e.g., pipe, cigar) was not assessed in the HINTS survey.

#### 2.1.5. Statistical Analyses

First, we conducted univariate analyses to describe the sample. We then performed multinomial logistic regressions to examine associations between location (California vs. remaining states), survey year (2017, 2019, 2020), and alcohol knowledge, controlling for covariates. Polynomial trend tests (for linear and quadratic trends), for those answering ‘Yes’ over survey years, were also conducted. We performed further multinomial logistic regressions to test the interaction between location and survey year and its association with alcohol/cancer knowledge (‘Yes’-main outcome of interest), adjusting for covariates. Predicted marginals were computed to obtain adjusted prevalence estimates of knowledge levels, which were compared across years and by location. We also performed a sensitivity analysis using multinomial logistic regression contrasting alcohol and cancer knowledge between California and its bordering states (Arizona, Oregon, and Nevada) and non-bordering states. Results showed no significant association between knowledge and this three-category location variable (*p* = 0.16). This suggested a lack of “spillover” effects related to P65, and we therefore focused on comparing California and the rest of the US.

For all analyses, final sample weights and jackknife replicate weights were used to obtain population-level point estimates and appropriate variance estimates. For HINTS 5 Cycle 3 (2019), we tested for group differences in the main outcome between mail and web respondents, no statistical differences were found; therefore, we used the combined sample and weights for cycle 3. Male and female gender identities were included in the model; however, because non-cisgender identities were only assessed in cycle 4, and because of the lack of accuracy and precision inherent to small cell sizes, people who identified with non-cisgender identities were excluded from the analysis.

## 3. Results

Demographic and cancer-related variables differed between California and the other states (Table 1). Namely, California participants were more racially/ethnically diverse than the other states (e.g., 37.5% Hispanic in California in 2020 vs. 13.2% in remaining states), reflecting California census estimates [25]. Moreover, California participants had a lower prevalence of cigarette/e-cigarette use than the other states (e.g., 11.2% in California in 2020 vs. 19.3% in remaining states) (Table 1) and more non-drinkers (50.7% vs. 47.9%) and fewer risky drinkers (17.4% vs. 23.5%) than the other states (Table 2). 

### 3.1. Alcohol/Cancer Knowledge Levels by Location and Year

#### 3.1.1. Location

In the model, testing location and survey year were included as main effects (no interaction term), differences by location were significant for California versus other states (*p* = 0.04). The adjusted prevalence of the knowledge of an association between alcohol and cancer risk (Table 3) was higher in California (41.6% (95% CI = 35.5–48.0%)) than the remaining states (34.1% (95% CI = 32.4–35.7%)). The adjusted prevalence of ‘Don’t know’ was slightly higher in the remaining states (34.1% (95% CI = 32.4–35.9%) than in California (31.7% (95% CI = 26.8–36.9%)). The adjusted prevalence of ‘No’ was higher in the remaining states (31.8% (95% CI = 30.2–33.4%)) than in California (26.7% (95% CI = 22.6–31.3%)).

#### 3.1.2. Survey Year

Differences in alcohol knowledge by survey year were also significant (*p* < 0.001). Overall, awareness of the alcohol-cancer link declined from 38.0% (95% CI = 34.6–41.6%) in 2017 to 32.6% (95% CI = 29.8–35.5%) in 2020 (Table 3). Linear and quadratic terms were both significant (*p* < 0.001) for alcohol and cancer knowledge (‘Yes’ answers). The latter reflects the curvilinear decline in people reporting awareness of this association. The percentage of ‘No’ responses declined sharply from 2017 to 2020 from 35.9% (95% CI = 32.8–39.1%) to 19.3% (95% CI = 17.1–21.8%), and ‘Don’t know’ responses increased from 26.1% (95% CI = 23.4–28.9%) in 2017 to 48.1% (95% CI = 45.2–50.9%) in 2020.

#### 3.1.3. Interaction between Location and Year

**Figure 1:**
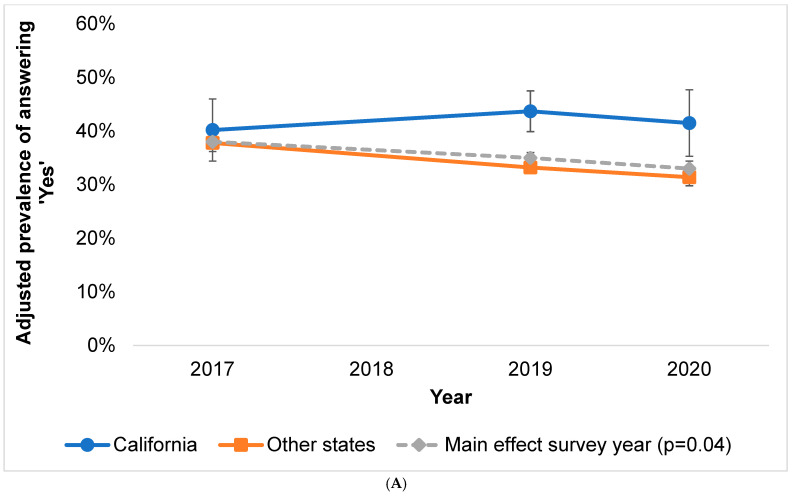

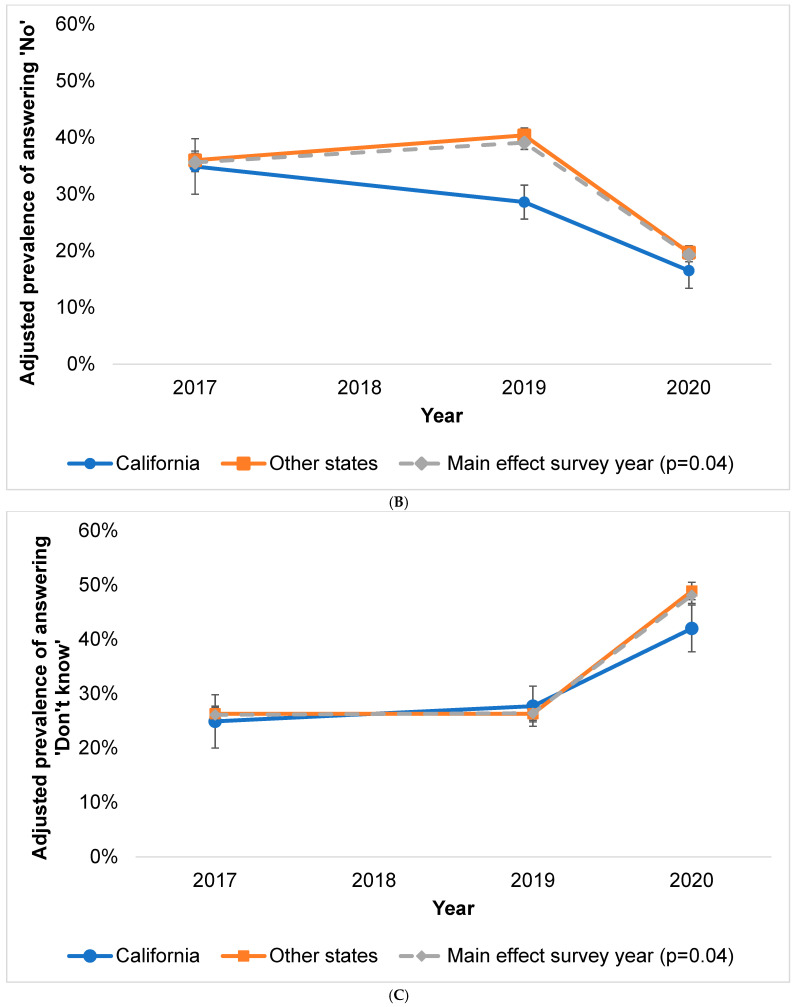
(**A**–**C**) Predicted marginals (adjusted prevalence) of alcohol and cancer knowledge responses in survey year*location interaction model (*p* = 0.32) with survey year main effect shown for comparison. (**A**) Adjusted prevalence of answering ‘Yes’ to alcohol and cancer knowledge item. (**B**) Adjusted prevalence of answering ‘No’ to alcohol and cancer knowledge item. (**C**) Adjusted prevalence of answering ‘Don’t know’ to alcohol and cancer knowledge item (error bars represent 95% Cis).

In California, ‘Yes’ responses were 40.2% in 2017, 43.7% in 2019, and 41.5% in 2020. ‘Yes’ responses in the remaining states were 37.8% in 2017, 33.2% in 2019, and 31.4% in 2020 (Figure 1). The proportion of the sample answering ‘No’ seemed to decline in both locations from 2019 to 2020, and the proportion responding ‘Don’t know’ seemed to increase in both locations. However, the interaction between location and year was not statistically significant (p_interaction_ ‘Yes’ = 0.32).

## 4. Discussion

This study examined alcohol and cancer knowledge in California versus the rest of the US in relation to changes in P65, the California law mandating warning signs at physical and online alcohol points of sale. We found that alcohol and cancer knowledge was higher in California than other states and that knowledge decreased overall across survey years. Although the difference in knowledge levels between California and the other states was greater in 2019 (13.1%) and 2020 (11.6%) than in 2017 (4.5%), suggesting an interaction between survey and location, this result was not statistically significant. One reason that we did not detect differences may be statistical power. An analysis showed that there was insufficient sample size to achieve 80% power in detecting an interaction between location and survey year. A second potential explanation is that the 2018 warning sign updates were modest in scope.

As predicted, we found that alcohol and cancer knowledge was higher in California than in the remaining states, which could reflect the presence of P65 warning signs since its establishment in 1986. Alternatively, characteristics of the California population and policy landscape may have led to a greater awareness of the health effects of alcohol. Enactment of Proposition 65 in California and other pioneering legislation related to a variety of other health topics along with lower rates of tobacco use in California support the idea that California tends to be more health aware than other states regardless.

Despite finding higher knowledge levels overall in California, there did not appear to be an effect from the 2018 P65 updates. One possible explanation for this is that the updated signage may not have been sufficiently strong to catch people’s attention and lead to changes in awareness. Replacing the existing warning signs with updated signs was hypothesized to bring renewed attention to the warnings, and the information on the P65 website may serve to increase the impact of the information on the warning signs. Including a web link may also reach people purchasing alcohol online. However, the 2018 update lacked changes to aesthetic characteristics, size, or specific messages about cancer, factors that appear to influence the effects of labeling in one recent study [26].

Previous studies of point-of-sale interventions with other behaviors may provide additional insight. For example, tobacco and sugary drink warnings have used pictorial warning signs [16,17,18,19] or warnings containing information about the risk of specific cancers [17,18]. For point-of-sale warnings concerning sugar sweetened beverages (SSBs), a recent review [27] of 5 experimental studies, largely in English speaking countries, concluded that such warnings are associated with reduced sales. However, none of the five studies examined the effects of such warnings on health-related knowledge and attitudes about SSBs. Unlike many past examples of labels and point of sale warnings, the P65 warning was text-only and lacked information about specific cancer types. For tobacco warnings, there is much more research concerning warning labels on packaging than on point-of-sale signage [28]. Nevertheless, the effectiveness of point-of sale marketing for tobacco and alcohol suggests that point-of-sale counter-marketing should be investigated further. Some studies of sugary drink or tobacco warnings also examined data collected during mass media campaigns intended to raise awareness of the risks of consuming those products [16,19], which can reinforce warning content [11]. The P65 changes were not, to our knowledge, accompanied by a formal media campaign.

Knowledge of the association between alcohol and cancer risk appears to remain low nationwide (~35–45%) [6,7]. This is troubling, considering that alcohol use is highly prevalent in the US, with 86% of adults reporting having ever used alcohol, and 55% currently using alcohol [29]. The effects of the original P65 warnings may have diminished over time. One study of the recall of cancer-related warning labels on alcohol beverages showed a decline in the recall of such messages from 24% to 13% in nationally representative samples of ~1000–2000 people between 1990 and 1994 in the US [11]. However, a lack of long-term surveillance of the awareness of a link between alcohol and cancer precludes further testing of the effects of signage and labeling. Past studies summarized by the World Health Organization have demonstrated that the most effective way to reduce alcohol consumption is through policies that limit access to alcohol, for example by increasing cost or decreasing hours open [30]. Therefore, in addition to studying point-of-sale warnings per se, it is important to study the interaction between alcohol warnings and access policies to understand their joint effectiveness for reducing alcohol use [31].

### Limitations

This is one of the first studies to evaluate the 2018 P65 alcohol warning updates, but with several limitations. First, sampling for HINTS was not conducted at the state level, and sample sizes for California were modest, limiting statistical power to detect an interaction between location and survey year. Also, because alcohol use behaviors may be associated with exposure to alcohol warnings [11], the inability to account for alcohol use in our models may have influenced our findings. Additionally, to our knowledge, data concerning alcohol and cancer knowledge are not available prior to the establishment of P65, making it impossible to determine if P65 signage or a persistent higher level of awareness of this link prior to the signage could explain the difference in awareness between California and other states. We also note that the requirement for revised signage was not made operative until August 30th, 2018 but was adopted in 2016 and businesses had the option of adopting the new language. We do not now how many businesses made this change prior to 2018.

A further limitation involves the change in wording of the alcohol knowledge question across HINTS cycles. This change was associated with an increased proportion of ‘Don’t know’ responses, potentially influencing our estimates of knowledge levels after the P65 update. Increases in ‘Don’t know’ responses could be a consequence of revising the item to ask about beer, wine, and liquor separately, perhaps suggesting to participants that there are differences of which they are unaware. Some evidence suggests that ‘Don’t know’ answers to survey items on health risks result from a lack of knowledge on a topic [32,33] but may also result from cognitive fatigue associated with processing complex survey items [34]. Lastly, while the survey response rates for the data used in our analyses were relatively low, these rates are consistent with other health surveys and reflects a downward trend in these rates [35]. A recent nonresponse study using HINTS [36] showed a slightly higher amount of health information seeking than seen in other health surveys and that incorporating the weights can compensate for any demographic bias that may be introduced.

## 5. Conclusions

This study demonstrates the potential for warning labels to affect drinking behavior. It also provides one of the few examples of research that evaluates a natural experiment between point-of-sale alcohol and cancer warnings as well as knowledge of the link between alcohol and cancer in the US. Further research evaluating potential effects of P65 and other alcohol and cancer warning policies on alcohol/cancer knowledge levels is needed [26,37]. Future studies could quantify media coverage of P65 changes and distinguish between people who purchase alcohol online versus in person. Finally, studies with larger samples would allow testing of potential associations between demographics and knowledge levels. Greater efforts are needed to determine how to increase awareness of the link between alcohol and cancer to help address the burden of alcohol-attributable cancers and increase support for policies aimed at reducing the harms of alcohol [8,9].

## Figures and Tables

**Table 1:** Sample characteristics of HINTS California and remaining states samples by year.

	HINTS Sample		California						Other States					
	All Years		2017		2019		2020		2017		2019		2020	
	N	% ^a^	N	% ^a^	N	% ^a^	N	% ^a^	N	% ^a^	N	% ^a^	N	% ^a^
**Total**	10,562	-	348	-	612	-	471	-	2394	-	3867	-	2870	-
**Aware of relationship between alcohol and cancer**														
Yes	3671	35.0	145	42.4	248	46.0	171	42.5	886	37.9	1358	32.9	863	30.9
No	2304	23.8	94	24.8	158	26.6	82	16.1	537	26.0	890	26.4	543	19.7
Don’t know	4587	41.2	109	32.9	206	27.3	218	41.4	971	36.1	1619	40.7	1464	49.3
**Gender**														
Male	4472	49.6	151	47.7	259	47.0	205	55.4	996	50.4	1659	49.7	1202	48.6
Female	6090	50.4	197	52.3	353	53.0	266	44.6	1398	49.6	2208	50.3	1668	51.4
**Age**														
18–39	2090	32.9	80	44.0	158	35.7	88	34.6	440	30.1	766	32.1	558	34.0
40–59	3581	40.9	141	36.2	202	42.7	163	40.3	856	43.5	1289	41.6	930	38.5
60+	4891	26.2	127	19.8	252	21.6	220	25.1	1098	26.4	1812	26.3	1382	27.5
**Education**														
High school or less	2409	28.7	75	29.5	123	25.5	116	25.5	566	28.4	845	28.6	684	29.7
Post high school/some college	3117	37.8	120	29.4	188	39.4	131	39.1	702	34.5	1129	40.2	847	39.4
College graduate or more	5036	33.5	153	41.1	301	35.1	224	35.4	1126	37.1	1893	31.2	1339	30.9
**Race**														
Black/African American	1535	11.4	26	5.1	54	6.3	42	8.3	365	11.6	589	12.2	459	12.5
White	7991	78.3	233	71.8	439	69.6	336	71.9	1834	79.8	2989	80.0	2160	78.2
Other/multiple	1036	10.3	89	23.1	119	24.1	93	19.8	195	8.7	289	7.8	251	9.3
**Hispanic ethnicity**														
Hispanic	1514	15.4	95	37.8	167	31.7	140	37.5	265	11.2	447	13.4	400	13.2
Not Hispanic	9048	84.6	253	62.2	445	68.3	331	62.5	2129	88.8	3420	86.6	2470	86.8
**Sexual orientation**														
Heterosexual	10,118	95.2	330	93.3	569	89.7	449	94.9	2304	95.4	3725	96.2	2741	95.1
Homosexual/gay/lesbian	253	2.7	12	4.4	25	6.7	13	2.9	51	3.0	90	1.9	62	2.4
Bisexual	191	2.1	6	2.3	18	3.6	9	2.2	39	1.6	52	1.9	67	2.6
**History of cancer**														
Yes	1680	8.9	42	6.1	75	6.2	71	8.9	381	9.0	640	9.4	471	8.9
No	8882	91.1	306	93.9	537	93.8	400	91.1	2013	91.0	3227	90.6	2399	91.1
**Family cancer history**														
Yes	7668	70.5	232	61.1	449	70.1	321	62.9	1753	71.4	2802	70.7	2111	71.9
No	2211	22.5	95	30.4	125	23.3	112	28.2	526	23.0	813	22.2	540	20.4
Not sure	683	7.0	21	8.4	38	6.6	38	8.9	115	5.6	252	7.1	219	7.8
**Smoking/e-cigarette use**														
Current smoker/user	1421	17.4	35	14.3	67	12.0	46	11.2	355	18.6	525	16.1	393	19.3
Not current smoker/user	9141	82.6	313	85.7	545	88.0	425	88.8	2039	81.4	3342	83.9	2477	80.7

^a^ Weighted percentages.

**Table 2:** Alcohol use among HINTS 5 cycle 4 participants.

	Total HINTS Sample		California		Other States	
	N	% ^a^ (SE) ^b^	N	% ^a^ (SE)	N	% ^a^ (SE)
**Non-drinking**	1631	48.2 (1.8)	220	50.7 (5.0)	1411	47.9 (1.8)
**Some drinking**	834	23.8 (1.2)	125	24.3 (3.8)	709	23.7 (1.3)
**Risky drinking**	671	22.8 (1.0)	91	17.4 (3.3)	580	23.5 (1.1)
**Missing**	205	5.1 (0.6)	35	7.6 (2.2)	170	4.8 (0.6)

^a^ Weighted percentage, ^b^ Standard error

**Table 3:** Predicted marginals of alcohol and cancer knowledge ^a^ estimated from logistic regression models ^b^.

	Yes	No	Don’t Know
	Adjusted Prevalence (%) (95% CI)	Adjusted Prevalence (%) (95% CI)	Adjusted Prevalence (%) (95% CI)
**Survey year**			
2017	38.0 (34.6–41.6)	35.9 (32.8–39.1)	26.1 (23.4–28.9)
2019	34.5 (32.5–36.5)	39.1 (36.7–41.5)	26.4 (23.9–29.1)
2020	32.6 (29.8–35.5)	19.3 (17.1–21.8)	48.1 (45.2–50.9)
**Location**			
California	41.6 (35.5–48.0)	26.7 (22.6–31.3)	31.7 (26.8–36.9)
Other states	34.1 (32.4–35.7)	31.8 (30.2–33.4)	34.1 (32.4–35.9)
**History of cancer**			
Yes	37.2 (33.1–41.4)	28.8 (25.5–32.4)	34.0 (30.2–38.1)
No	34.8 (33.1–36.5)	31.4 (29.9–33.0)	33.8 (32.1–35.5)
**Family cancer history**			
Yes	36.2 (34.1–38.3)	31.1 (29.3–33.0)	32.7 (30.8–34.6)
No	33.9 (30.7–37.2)	30.9 (27.6–34.5)	35.2 (31.7–38.8)
Not sure	26.3 (19.7–34.2)	32.9 (26.7–39.8)	40.8 (33.2–48.9)
**Gender**			
Male	35.7 (33.2–38.3)	30.8 (28.4–33.4)	33.5 (30.9–36.2)
Female	34.3 (32.2–36.4)	31.6 (29.7–33.4)	34.2 (32.3–36.0)
**Cigarette/e-cigarette use**			
Current smoker/user	31.3 (27.7–35.2)	32.0 (27.8–36.7)	36.7 (31.9–41.6)
Not current smoker/user	35.8 (33.9–37.7)	31.0 (29.4–32.7)	33.2 (31.5–35.0)
**Age group**			
18–39	38.8 (35.2–42.4)	25.0 (21.4–28.9)	36.3 (32.8–39.9)
40–59	34.4 (31.9–36.9)	32.7 (30.6–34.9)	32.9 (30.3–35.6)
60+	31.2 (29.1–33.3)	36.5 (34.2–38.8)	32.3 (30.3–34.5)
**Education**			
High school degree or less	29.4 (26.4–32.6)	33.1 (30.2–36.0)	37.6 (34.4–40.8)
Post high school/some college	34.1 (31.0–37.4)	32.0 (29.3–34.7)	33.9 (31.1–36.9)
College graduate or more	40.5 (38.1–43.0)	28.8 (26.7–31.0)	30.6 (28.4–32.9)
**Race**			
White	34.8 (33.0–36.6)	31.4 (29.8–33.1)	33.8 (32.0–35.6)
Black/African American	29.2 (25.4–33.3)	32.8 (28.7–37.1)	38.1 (33.4–43.0)
Other/multiple	42.8 (37.4–48.4)	27.7 (23.7–32.1)	29.5 (25.2–34.2)
**Hispanic ethnicity**			
Hispanic	33.3 (29.0–37.8)	32.1 (28.2–36.3)	34.6 (30.2–39.4)
Not Hispanic	35.3 (33.6–37.1)	31.0 (29.4–32.7)	33.7 (31.9–35.4)
**Sexual orientation**			
Heterosexual	34.7 (33.0–36.4)	31.4 (29.9–33.0)	33.9 (32.3–35.5)
Homosexual/gay/lesbian	41.1 (32.1–50.6)	25.6 (17.4–36.0)	33.3 (23.5–44.8)
Bisexual	40.8 (29.3–53.5)	26.7 (14.8–43.2)	32.5 (19.5–48.9)

^a^ Items asks, “In your opinion, how much does drinking the following types of alcohol affect the risk of getting cancer?” ^b^ Survey weighted logistic regression models.

## Data Availability

HINTS data are available for download here—https://hints.cancer.gov (accessed on 13 September 2022). There is an application process for the use of state level identifiers.
